# VUV Photofragmentation of Chloroiodomethane: The Iso-CH_2_I–Cl and Iso-CH_2_Cl–I Radical Cation Formation

**DOI:** 10.1021/acs.jpca.0c05754

**Published:** 2020-08-12

**Authors:** Anna Rita Casavola, Antonella Cartoni, Mattea Carmen Castrovilli, Stefano Borocci, Paola Bolognesi, Jacopo Chiarinelli, Daniele Catone, Lorenzo Avaldi

**Affiliations:** † Institute of Structure of Matter-CNR (ISM-CNR), Area della Ricerca di Roma 1, Via Salaria km 29.300, 00015 Monterotondo, Italy; ‡ Department of Chemistry, Sapienza University of Rome, P.le Aldo Moro 5, 00185 Rome, Italy; § Department for Innovation in Biological, Agrofood and Forest Systems, University of Tuscia, Viterbo 01100, Italy; ∥ Institute for Biological Systems-CNR (ISB-CNR), Area della Ricerca di Roma 1, Via Salaria, Km 29.500,, 00015 Monterotondo, Italy; ⊥ Institute of Structure of Matter-CNR (ISM-CNR), Area della Ricerca di Tor Vergata, Via del Fosso del Cavaliere, 00133 Rome, Italy

## Abstract

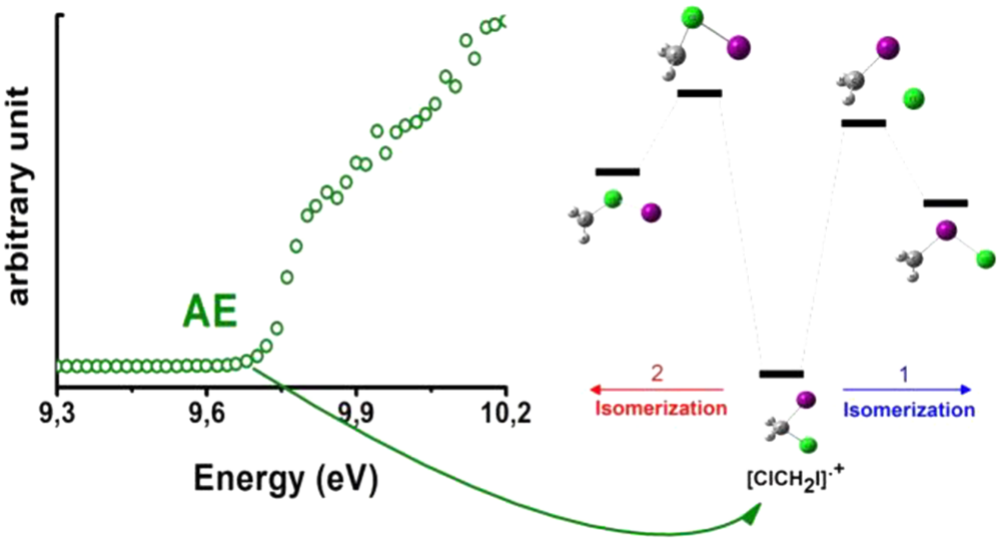

Dihalomethanes XCH_2_Y (X and Y = F, Cl, Br, and I) are a class of compounds involved in several processes leading to the release of halogen atoms, ozone consumption, and aerosol particle formation. Neutral dihalomethanes have been largely studied, but chemical physics properties and processes involving their radical ions, like the pathways of their decomposition, have not been completely investigated. In this work the photodissociation dynamics of the ClCH_2_I molecule has been explored in the photon energy range 9–21 eV using both VUV rare gas discharge lamps and synchrotron radiation. The experiments show that, among the different fragment ions, CH_2_I^+^ and CH_2_Cl^+^, which correspond to the Cl- and I-losses, respectively, play a dominant role. The experimental ionization energy of ClCH_2_I and the appearance energies of the CH_2_I^+^ and CH_2_Cl^+^ ions are in agreement with the theoretical results obtained at the MP2/CCSD(T) level of theory. Computational investigations have been also performed to study the isomerization of geminal [ClCH_2_I]^•+^ into the iso-chloroiodomethane isomers: [CH_2_I–Cl]^•+^ and [CH_2_Cl–I]^•+^.

## 1 Introduction

Dynamical processes triggered by molecular photofragmentation are relevant in different fields, spanning from the solar energy conversion to medical applications.^1−6^ Halomethanes are species with a great impact on the chemistry of the Earth’s atmosphere. They influence the HOx and NOx cycles involved in catalytic ozone depletion,^7,8^ participate to the marine and coastal aerosol formation which affects Earth’s radiation balance,^9^ and alter the oxidative capacity of the atmosphere reacting with OH. Moreover the neutral fragment CH_2_I, obtained by photolysis of CH_2_I_2_, produces the simplest “Criegee intermediate”, CH_2_OO, in the reaction with oxygen O_2_ in laboratory studies.^10,11^ Aerosols could also act as cloud condensation nuclei (CCN), which further form clouds to influence the radiation balance of the globe.^12^ Many efforts have been devoted to understanding the source of these species, the dynamics of their photofragmentation processes, and the role of their decomposition products in the chemistry of Earth’s atmosphere. However, many aspects, like for instance the pathways leading to species such as HIO_3_ and iodine aerosol formation, are not entirely understood.^13^ It is well-known that the study of atmospheric chemistry is mainly based on neutrals, which are the most abundant species present in the environment. However, also ion chemistry plays a role in environmental processes. Indeed, ions produced by human activity or natural events (i.e., corona discharge, lightning, high voltage power lines, hot surfaces, and cosmic rays) quickly react with the surrounding molecules and produce new ionic and neutral species, in turn involved in the atmospheric reactivity.^14−16^ It is also clear that charged species are involved in aerosol formation and can affect the Earth’s climate.^17^ It is therefore fundamental to understand the link between ion and neutral chemistry. As for the halo-compounds and halomethanes, dihalomethanes XCH_2_X or XCH_2_Y (where X and Y= I, Br, Cl and F) have been largely studied,^18−24^ because for example the daytime destruction of O_3_ over coastal areas is mainly due to I atoms,^25^ while Br and Cl atoms are mainly involved in the reaction with VOCs.^26^ The iodine-containing dihalomethanes are mainly emitted by oceans^27^ and consist of volatile organic iodine compounds (VOICs): CH_2_I_2_, ClCH_2_I and BrCH_2_I, CH_3_I with lifetimes in the range from minutes (CH_2_I_2_) to days (CH_3_I). Bromine and chlorine halomethanes have a longer lifetime and, hence, are more equally distributed in the atmosphere and may play a significant role in the higher atmosphere.

**Scheme 1:**
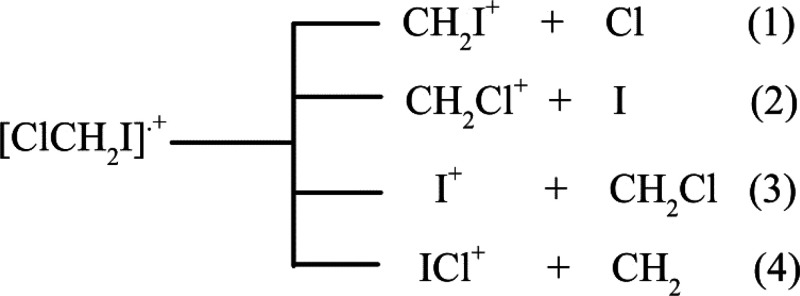
Main Fragmentation Channels from [ClCH_2_I]^•+^

In this work we focused on the VUV photofragmentation of chloroiodomethane, ClCH_2_I, a VOIC present in the environment due to marine microalgae^28^ and volcanic activity.^29^ Its photochemistry is a source of iodine atoms which are further involved in several chemical physics processes.^30,31^ It has been reported that condensed-phase photolysis of XCH_2_Y geminal species induces a halogen shift leading to iso-halomethane isomers where a halogen–halogen bond is formed (CH_2_X-Y).^32,33^ Reid et al.^34,35^ demonstrated that this isomerization in the gas phase is a pathway to molecular products (XY) comparable to the simple C-halo bond fission. Other works demonstrate that iso-dihalomethanes are reactive photointermediates in solution leading to the cyclopropanation of alkenes.^36−38^ The neutral ClCH_2_I molecule has been extensively studied,^39−43^ but to the best of our knowledge, less information exists for the radical ion. In particular, the photofragmentation of ClCH_2_I has been investigated in the energy range 10–13 eV with the threshold photoelectron photoion coincidence (TPEPICO) technique.^44^ Here, we explore the interaction of VUV radiation with an effusive beam of ClCH_2_I in the photon energy range 9–21 eV. The photofragmentation mass spectra at the different photon energies of a rare gas discharge lamp have been acquired and the appearance energy (A*E*_exp_) of selected fragments has been measured with tunable synchrotron radiation and compared with theoretical ones (A*E*_th_). The formation of iso-dihalomethanes [CH_2_I–Cl]^•+^ and [CH_2_Cl–I]^•+^ has been also explored by *ab initio* calculations, and a comparison with [CH_2_I–I]^•+^ has been carried out.^19^ The main channels considered in the experiments are reported in Scheme 1.

## 2 Experimental Methods

### 2.1 VUV Rare Gas Discharge Lamp Experiments

The discharge lamp was operated with a gas pressure in the discharge chamber that varies from 10^–1^ to 10^–3^ mbar, depending on the rare gas, and a driving current of 5 mA. The main lines,^45^ in the three used gases, are at 21.22 eV (He I), 16.67 eV (Ne I), and 11.62 eV (Ar I). The output radiation is not monochromatized; hence, an unknown contribution of wavelengths different from the main lines listed above cannot be excluded.^46^ The effusive beam of the target molecule ClCH_2_I is brought to the interaction region via a gas inlet, and the base pressure in the experimental chamber is about 3 × 10^–8^ mbar. The ions produced by the interaction of the photon beam with the target molecules are extracted from the interaction region by a 700 V/cm DC electric field and accelerated into a Wiley–McLaren time-of-flight (TOF)^47^ analyzer. The flight time measurement for mass/charge analysis is triggered by the detection of a kinetic energy unresolved photoelectron via a channeltron electron multiplier mounted opposite to the TOF spectrometer. The scheme of the setup and other technical details are reported in previous works^18^ and will be not repeated here. The intensities of the parent ion, [ClCH_2_I]^•+^, and of the main fragments, I^+^, CH_2_I^+^, and CH_2_Cl^+^, have been calculated as the integrated yield over each peak in the mass spectrum. The intensities of these fragments have then been used to calculate the respective branching ratio as a function of photon energy.

### 2.2 Synchrotron Experiments

The measurements of the A*E*_exp_ of the ionic fragments obtained from the photofragmentation of ClCH_2_I were carried out at the “Circular Polarization” (CiPo) beamline^48^ of the Elettra synchrotron radiation source (Trieste, Italy), which is fed by an Electromagnetic Elliptical Wiggler. The VUV radiation was monochromatized by an aluminum normal incidence monochromator (Al-NIM) that covers the photon energy range 5–17 eV with a resolving power of about 1000. The experimental apparatus is described in detail elsewhere,^49,50^ and only a brief description will be reported here. It consists of an ionization region where the target molecules are admitted through a needle valve which regulates the gas flow of the effusive beam. Ion optics extract and focus the photoions into a commercial quadrupole mass spectrometer equipped with a channeltron detector. The photoionization efficiency curves (PIECs) of the selected ions are obtained by reporting the yield of the selected ions versus photon energy (9–16 eV), scanning both the wiggler and the monochromator in order to have the maximum flux at each given energy. The energy step and the acquisition time were 20 meV and 10–20 s/point, respectively. The PIECs were normalized to the photon intensity, measured by a photodiode located after the interaction region. A lithium fluoride filter was used below 11.7 eV to remove the higher order radiation contribution; above this energy the higher order radiation contribution was evaluated by comparing the Ar^+^ ion yield measured as a function of the photon energy to its ionization cross-section.^51^ The photon energy was calibrated against the autoionization features observed in the Ar^+^ photoionization efficiency spectrum between the 3p spin–orbit components. The A*E*_exp_ values are determined by fitting the PIECs, plotted on a linear scale, by two straight lines representing the background and the ion signal in the threshold region, respectively. The photon energy at the intersection of these two lines is the experimental determination of the A*E*_exp_. For each detected ion, the fitting procedure has been repeated considering increasing ranges in the threshold region as long as a reproducible A*E*_exp_ value could be determined.^49^ The average of all these results gives the A*E*_exp_ and its uncertainty, estimated to be in the range 10–190 meV, depending on the shape of the PIEC onset. It is important to remember that the experimental value of the A*E*_exp_ also depend on the sensitivity of the experimental setup (time of acquisition, number of counts, statistics, efficiency and lifetime of the ions), so that they have to be considered as upper limits of the effective A*E*_exp_. For sake of comparison, a mass spectrum has been also measured at the photon energy of 11.5 eV, and the relative intensity of the peaks have been obtained with the same procedure used for the spectra acquired with the gas discharge lamp setup.

Liquid ClCH_2_I was purchased from Sigma-Aldrich with purity higher than 97%. The vapor pressure of the compound allows to keep the sample in a test tube outside the chamber. Several freeze–pump–thaw cycles were performed on the sample.

## 3 Theoretical Methods

The geometries of interest have been optimized at the MP2 level using 6-311++G** basis set for the C, Cl, and H atoms. The small-core (28 electrons) scalar-relativistic effective potential (ECP-28) in conjunction with the aug-cc-pVTZ-PP basis set has been chosen for the iodine atom.^52,53^ MP2 was employed within the frozen-core approximation by using the 4s4p4d frozen-core orbitals for the iodine atom. Accurate total energies were obtained by single-point coupled cluster calculations, CCSD(T)^54^ using the same basis sets and pesudopotential for the MP2 calculations. CCSD(T) have been performed in full mode (Table S1 in the SI). All critical points were characterized as energy minima or transition structures (TS) by calculating the corresponding MP2 harmonic frequencies, also used to evaluate the zero-point energy correction. The TS were unambiguously related to their interconnected energy minima by intrinsic reaction coordinates (IRC) calculations.^55,56^ Small spin contamination was revealed, at the MP2 level of theory, in radicals and radical cations as indicated by the ⟨S^2^⟩ operator close to the theoretical value for the pure doublet spin state (0.75) or triplet state (2). The CCSD T1 diagnostic is within the recommended threshold of 0.02.^57^ This suggests that the wave functions of these species are correctly described by a single-reference method. The dissociation energy of the species **2b** [CH_2_I···Cl]^•+^ (Table S1), considered as a complex, was corrected for the basis set superposition error (BSSE) using the counterpoise method by Boys and Bernardi.^58^ The Mulliken analysis^59^ was used to compute the charge population in order to explore, in a qualitative way, the charge distribution. The validation of the calculations performed on the cationic ground state and without considering spin–orbit coupling^60^ have been discussed in our previous work on the photofragmentation of [CH_2_I_2_]^•+^,^19^ where, for instance, a good agreement between experimental value of the appearance energy of CH_2_I^+^ (Δ*E*_298_ = 10.42–10.55 eV NIST database^61^) and the theoretical one (10.51 eV) has been obtained. Neglecting spin–orbit coupling might be considered an approximation in processes involving iodine atom, however the agreement achieved between experimental and theoretical appearance energy of the ion I^+^ from [CH_2_I_2_]^•+^,^20^ the results of this work and previous studies on [ClCH_2_I]^•+^^44^ confirm that, in some photofragmentation processes involving iodine atom, spin–orbit effects can be neglected. The vertical ionization energies (I*E*) of the outer valence orbitals were also calculated using the outer valence Green function OVGF/6-311++G** methods.^62,63^ All calculations were performed using Gaussian 09.^64^ The adiabatic A*E*_th_ at 298 K has been calculated from well-established procedures.^49,65,66^ It is worth underlying that the theoretical values of appearance energy may be lower than their corresponding experimental values as the calculations could not consider transition states (TS, reverse activation barrier), kinetic shifts, unfavorable Franck–Condon factors, or possible excited states which may affect the fragmentation processes.

## 4 Results and Discussion

**Figure 1:**
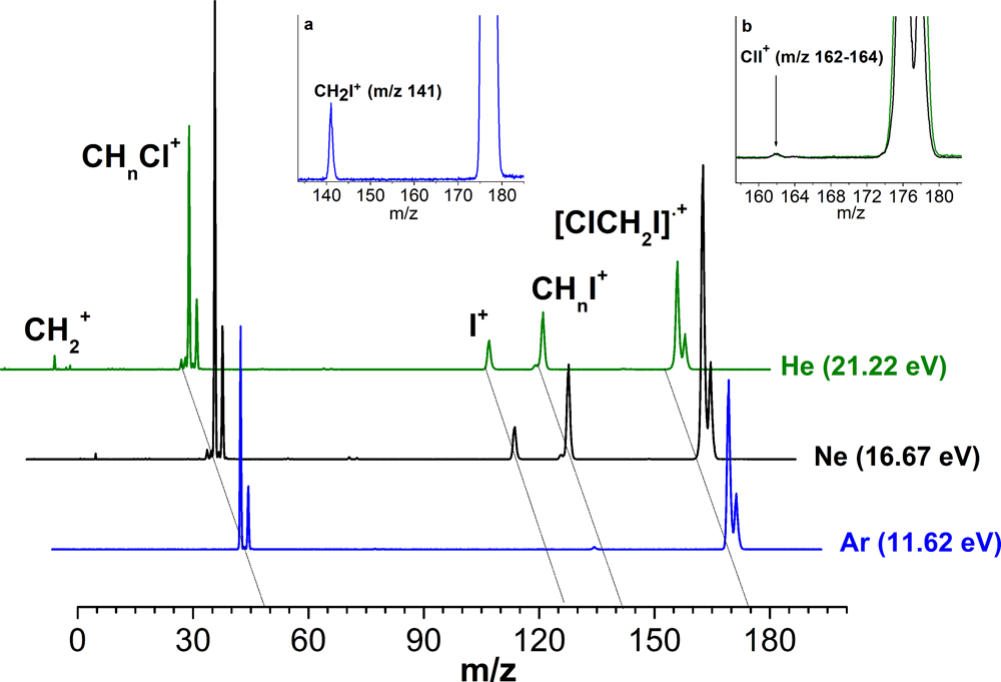
Mass spectra of ClCH_2_I (shown in offset mode) obtained with the He (green line), Ne (black line) and Ar (blue line) rare gas discharge lamps and their respective most intense photoemission line indicated in brackets. In the insets an enlarged view of *m*/*z* range 133–185 (a) and 158–183 (b) acquired with Ar (a), and Ne and He (b) lamps are shown. For CH_*n*_Cl^+^ and CH_*n*_I^+^, *n* = 2 to 0.

The mass spectra recorded with the VUV rare gas discharge lamps at 11.62 (Ar), 16.67 (Ne), and 21.22 eV (He) are shown in Figure 1. All species containing the Cl atom are characterized by a doublet peaks due to the two Cl isotopes. The data indicate that the main features in the mass spectra are the parent ion [ClCH_2_I]^•+^ (*m*/*z* = 176 and 178), the CH_2_I^+^ (*m*/*z* = 141), CH_2_Cl^+^ (*m*/*z* = 49 and 51), and I^+^ (*m*/*z* = 127) ions produced in fragmentations (1) to (3) of Scheme 1. The tiny feature observed at *m*/*z* = 162 and 164 (panel b of Figure 1) is attributed to the ICl^+^ ions (path 4 of Scheme 1).

Traces of CH_2_^+^, CCl^+^, CI^+^, CHCl^+^, and CHI^+^ species are also detected in the mass spectrum measured with He lamp. A small peak at *m*/*z* = 18 is observed with Ne and He lamps (Figure 1) due to the presence of water trace in the ion source. At the lowest photon energies of 11.62 eV only the fragment at *m*/*z* = 49 and 51 (CH_2_Cl^+^) is observed. In Table 1 the fragments branching ratios are reported together with those obtained with synchrotron radiation at 11.5 eV. These data show that, while the relative intensity in percentage of the CH_2_Cl^+^ ions remains almost constant for increasing photon energy, the relative contribution of the parent ion [ClCH_2_I]^•+^ and of the others fragments respectively decreases and increase. This clearly indicates how the generation of these fragments can be directly connected to the parent ion fragmentation. Moreover, the mass spectra show that the CH_2_Cl^+^ (*m*/*z* = 49 and 51) has an A*E*_exp_ lower than 11.62 eV, while all of the other ions have an A*E*_exp_ higher than 11.62 eV or around this value since a trace of the CH_2_I^+^ (*m*/z = 141) is already observed at 11.62 eV photon energy (panel a of Figure 1).

**Table 1:** Branching Ratios (Relative Intensities in Percentage, %) among CH_2_Cl^+^, I^+^, CH_2_I^+^, and [ClCH_2_I]^•+^ from Fragmentation of the ClCH_2_I Molecule with Ar, Ne, and He Ionization Source and with Synchrotron Radiation at *h*ν 11.5 eV

	Ar (11.62 eV)	Ne (16.67 eV)	He (21.22 eV)	synchrotron 11.5 eV
CH_2_Cl^+^	48 ± 2	46 ± 2	48 ± 2	51 ± 2
I^+^		4 ± 2	8 ± 2	
CH_2_I^+^		7 ± 2	14 ± 2	
[ClCH_2_I]^•+^	52 ± 2	43 ± 2	30 ± 2	49 ± 2

**Figure 2:**
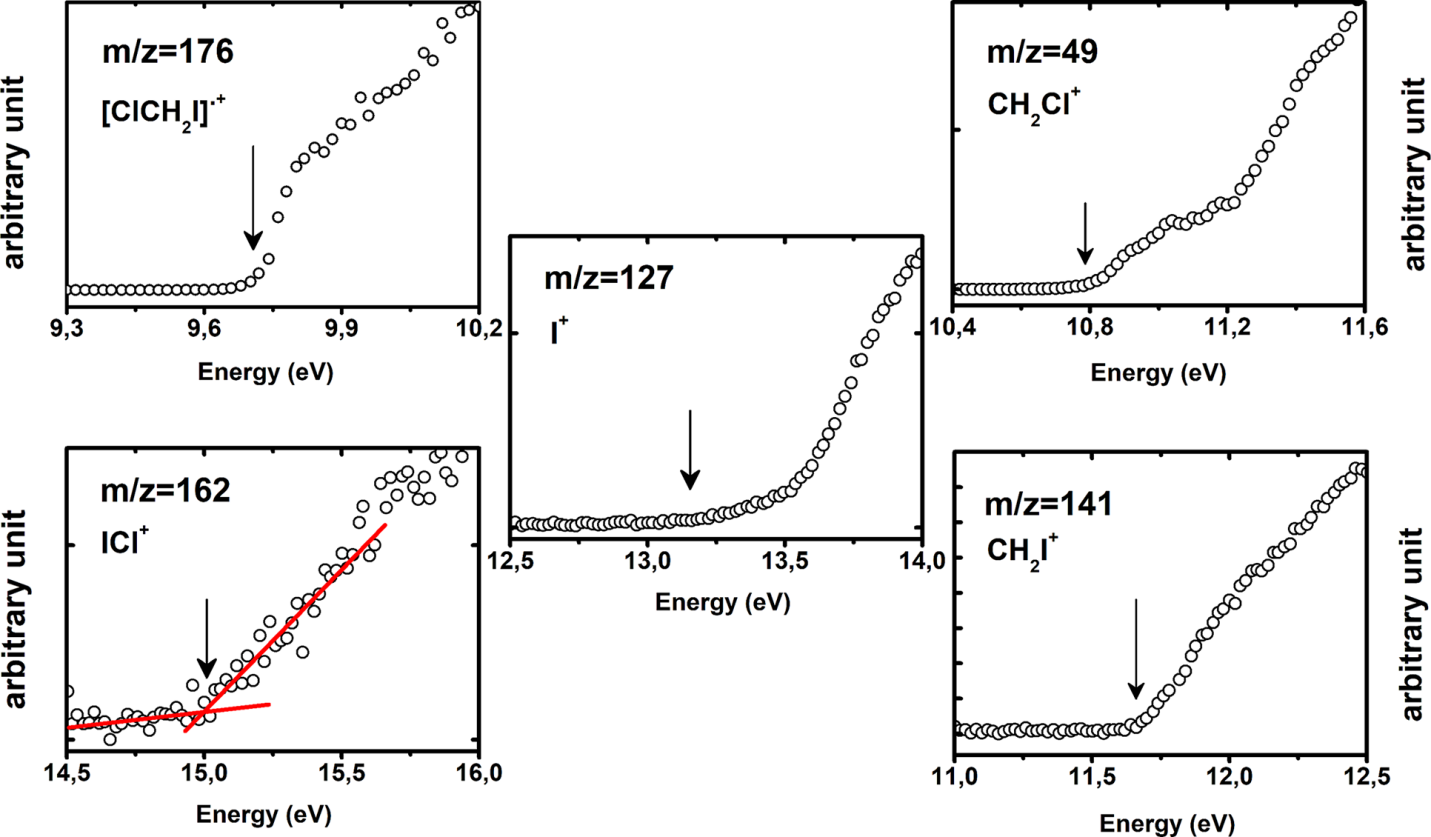
PIECs of the five selected ions [ClCH_2_I]^•+^ (*m*/*z* = 176), ICl^+^ (*m*/*z* = 162), I^+^ (*m*/*z* = 127), CH_2_I^+^ (*m*/*z* = 141), and CH_2_Cl^+^ (*m*/*z*= 49). The fitted A*E*_exp_ values are indicated by arrows and reported in Table 2.

The PIECs of the [ClCH_2_I]^•+^ (*m*/*z* = 176), ICl^+^ (*m*/*z* = 162), I^+^ (*m*/*z* = 127), CH_2_I^+^ (*m*/*z* = 141), and CH_2_Cl^+^ (*m*/*z* = 49) ions are shown in Figure 2, where the A*E*_exp_ values are indicated by an arrow. An example of the linear fit used to extract the A*E*_exp_ is shown in the case of the ICl^+^ ion. The experimental and theoretical values of the appearance energy from this work are reported in Table 2 where they are also compared with literature data.

**Table 2:** A*E*_exp_ and Adiabatic A*E*_th_ for the Main Fragment Ions from ClCH_2_I at 298 K

ClCH_2_I			
ions (*m*/*z*)	A*E*_exp_ (eV)	A*E*_exp_^a^ (eV)	A*E*_th_ (eV)
[ClCH_2_I]^•+^ (176)	9.71 ± 0.01	9.752 ± 0.012	9.70
CH_2_Cl^+^ (49) + I	10.79 ± 0.01	10.878 ± 0.010	10.87
CH_2_I^+^ (141) + Cl	11.66 ± 0.03	11.656 ± 0.030	11.46
I^+^ (127) + CH_2_Cl	13.15 ± 0.19		12.83
ICl^+^ (162) + CH_2_	15.01 ± 0.02		14.01
CI^+^ (139)	15.63 ± 0.01		
CCl^+^ (47)	14.88 ± 0.01		

a Reference (44).

The PIECs of CCl^+^ (*m*/*z* = 47) and CI^+^ (*m*/*z* = 139) ions has been also recorded and reported in Figure S1 of the SI. The [ClCH_2_I]^•+^ ion is generated in its ionic ground state by the ejection of an electron from the iodine nonbonding orbitals.^67^ The measured A*E*_exp_ of 9.71 ± 0.01 eV (Table 2 and Figure 2) is consistent with the previous values of 9.752 ± 0.012 and 9.7506 ± 0.0006 eV obtained by TPEPICO and mass-analyzed threshold ionization (MATI) experiments, respectively.^44,68^

**Figure 3:**
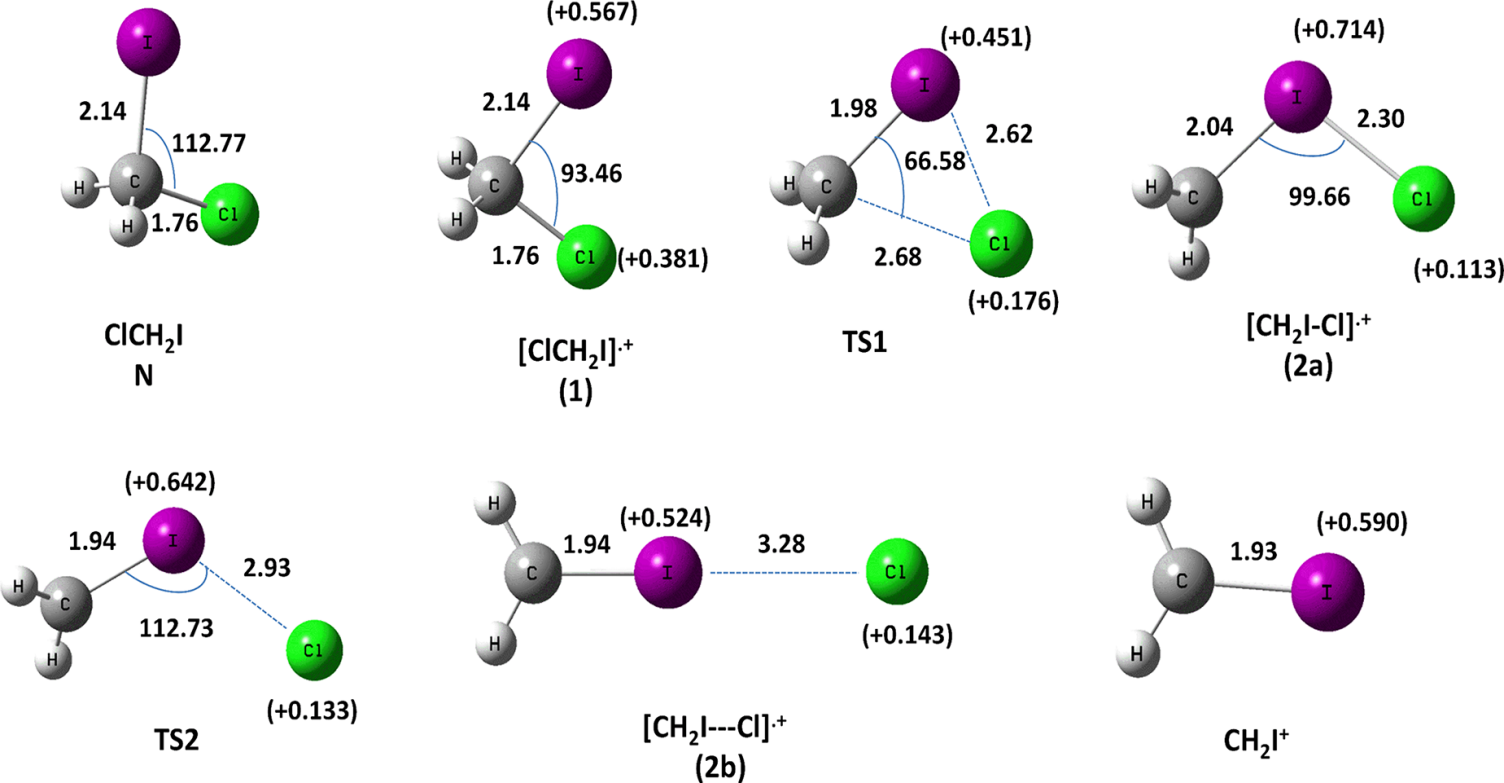
Optimized geometries (distances in Å and angles in degrees) calculated at the MP2 level and the Mulliken atomic charge, *e* (in brackets) on the I and Cl atoms of the species involved in the Cl-loss channel from [ClCH_2_I]^•+^ (see also Table S1).

The optimized geometries of the stationary points (minima and TS) of the ClCH_2_I and [ClCH_2_I]^•+^ potential energy surface (PES) are shown in Figures 3 and 5.

The geometrical parameters of the species ClCH_2_I (**N**) and [ClCH_2_I]^•+^ (**1**) in their ground states are also reported in Tables 3 and 4, respectively, and compared with DFT calculations (with and without spin–orbit effect)^69^ and experimental data.^70^

**Table 3:** Geometric Parameters of ClCH_2_I in the Electronic Ground State

	bond length (Å)				bond angles (deg)		
methods	C–Cl	C–I	C–H	Cl–C–I	Cl–C–H	I–C–H	H–C–H
experiments^a^	1.774	2.137	1.062	112.5	108.4	108.3	111.0
this work MP2 (see theoretical methods)	1.763	2.143	1.087	112.8	109.4	106.6	112.2
DFT/B3LYP^b^ (without spin–orbit effect)	1.778	2.181	1.081	114.4	108.6	106.6	112.1
(with spin–orbit effect)	1.777	2.187	1.081	114.4	108.7	106.6	112.0

a Reference (70).

b Reference (69).

**Table 4:** Geometric Parameters of [ClCH_2_I]^•+^ in the Ionic Electronic Ground State^a^

	bond length (Å)				bond angles (deg)		
methods	C–Cl	C–I	C–H	Cl–C–I	Cl–C–H	I–C–H	H–C–H
this work MP2 (see theoretical methods)	1.760	2.139	1.087	93.5 (147 cm^–1^)	112.3	109.9	116.5
DFT-B3LYP (without spin–orbit effect)^b^	1.767	2.187	1.082	96.1 (160 cm^–1^)	111.9	109.6	115.8
(with spin–orbit effect)^b^	1.738	2.242	1.084	106.0 (112 cm^–1^)	112.7	104.9	114.5

a In brackets the calculated vibrational frequency of the Cl–C–I bending mode is reported. The experimental value^71^ is 114 cm^–1^.

b Reference (69).

**Figure 4:**
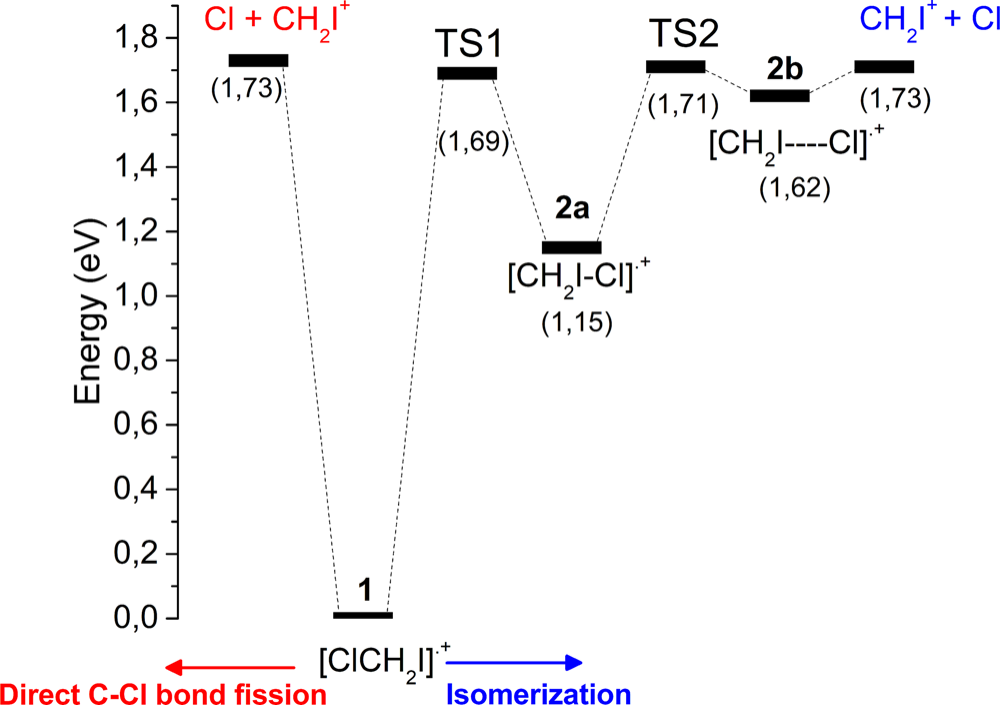
Potential energy profile calculated at the CCSD(T,full)//MP2 level of the theory for the dissociation of [ClCH_2_I]^•+^ radical cation (**1**) into CH_2_I^+^ + Cl. On the left (in red) the direct C–Cl bond fission and on the right (in blue) the dissociation through isomerization into [CH_2_I–Cl]^•+^ (**2a**). In brackets are reported the energies of the species relative to [ClCH_2_I]^•+^.

As reported in ref (69) the spin–orbit effect has a relevant influence mainly in the Cl–C–I bending frequency of the cation (see also Table S2 in SI) whose experimental value is 114 cm^–1^, while DFT and MP2 theories predict 160 and 147 cm^–1^, respectively (Table 4). These differences in the frequency reflect in the differences of the Cl–C–I bond-angle, with values of 93.5° (MP2) and 96.1° (DFT) with spin–orbit effect neglected and 106.0° when it is considered. However, as discussed in the theoretical methods, this difference does not affect substantially the calculation of several properties as, for instance, the adiabatic ionization energy of ClCH_2_I at 9.70 eV, which matches the experimental value of 9.71 ± 0.01 eV (see Table 2). Focusing on the Cl-loss channel (path (1) in Scheme 1), the A*E*_exp_ = 11.66 ± 0.03 eV (Figure 2) is in agreement with the calculated value of 11.46 eV and with previous data (Tables S1 and 2). No transition state has been found for the direct C–Cl bond breaking at variance with the same channel in the neutral ClCH_2_I, where a TS has been identified.^43^ As for the previous study of the I-loss channel from [ICH_2_I]^•+^ ions,^19^ the possibility of the isomerization of the geminal [ClCH_2_I]^•+^ into iso-dihalomethanes [CH_2_I–Cl]^•+^ and [CH_2_Cl–I]^•+^ before dissociation, has been explored by *ab initio* calculations. The optimized geometries of the minima (**1**, **2a**, and **2b**) found on the [ClCH_2_I]^•+^ PES, the transition states **TS1** and **TS2** connecting **1**-**2a** and **2a**-**2b**, respectively, and the optimized structure of the CH_2_I^+^ ion are shown in Figure 3. The energy profile calculated for the Cl-loss channel of [ClCH_2_I]^•+^ leading to CH_2_I^+^ and Cl is shown in Figure 4.

**Figure 5:**
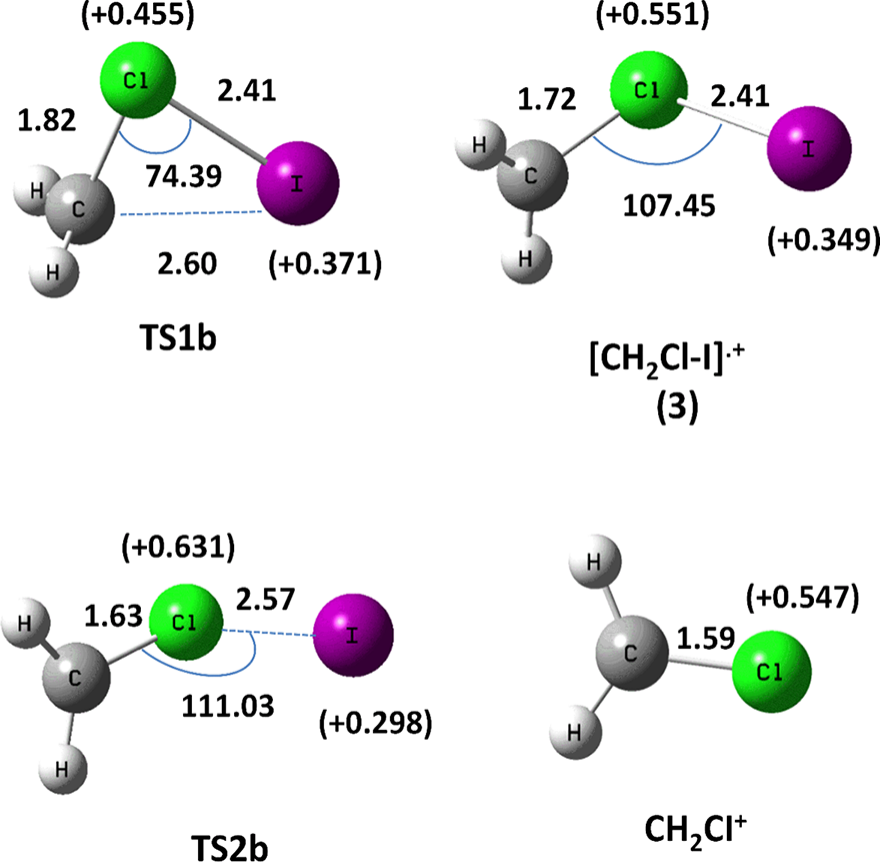
Optimized geometries (distances in Å and angles in degrees) calculated at the MP2 level of the theory and the Mulliken atomic charge, *e* (in brackets) on the I and Cl atoms of the species involved in the I-loss channel from [ClCH_2_I]^•+^ (see also Table S1).

**Figure 6:**
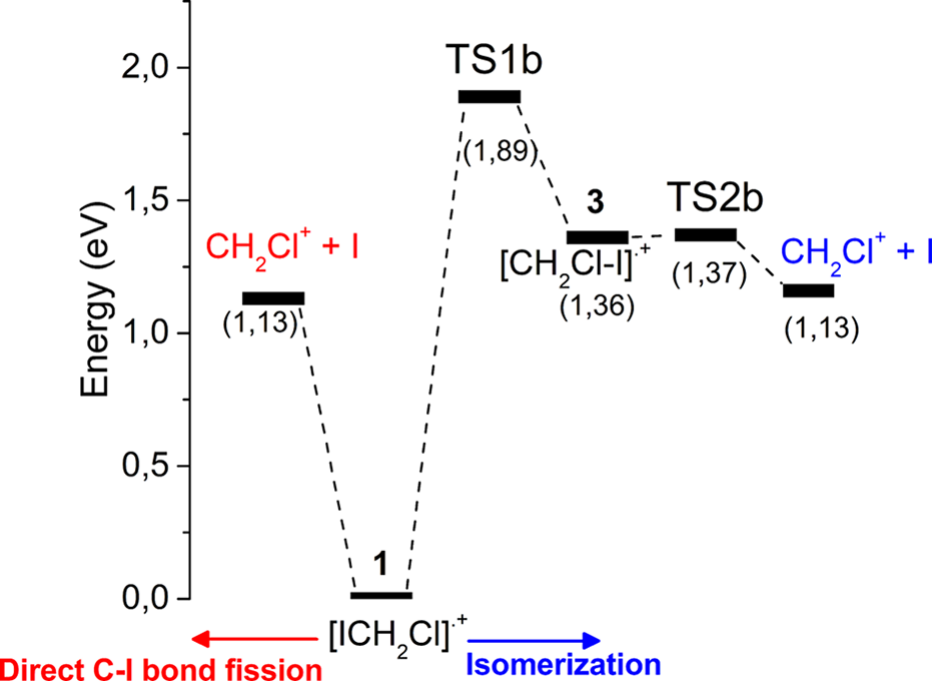
Potential energy profile calculated at the CCSD(T,full)//MP2 level of the theory for the dissociation of [ClCH_2_I]^•+^ radical cation (**1**) into CH_2_Cl^+^ + I. On the left (in red) the direct C–I bond fission and on the right (in blue) the dissociation through isomerization into [CH_2_Cl–I]^•+^ (**3**). In brackets are reported the energies of the species relative to [ICH_2_Cl]^•+^.

Two energetically competitive pathways that lead to the Cl and CH_2_I^+^ with the same A*E*_th_ (CH_2_I^+^) of 11.46 eV have been identified. The first route (on the left side of Figure 4) is the direct C–Cl bond breaking with a dissociation energy of 1.73 eV, while the second route (on the right side of Figure 4) is the isomerization of the geminal [ClCH_2_I]^•+^ radical cation **1** into the iso-isomer [CH_2_I–Cl]^•+^**2a** through **TS1** (imaginary frequency 576.6 i cm^–1^) at 1.69 eV (Figure 4). In isomer **2a** a halogen–halogen bond, I–Cl, is established, the charge is mainly located on the iodine atom (+0.714 *e*) and its energy is 1.15 eV higher than that of geminal cation **1**. A similar energy difference was found in diiodomethane, between the geminal [ICH_2_I]^•+^ and iso [CH_2_I–I]^•+^ radical cation isomers (0.97 eV).^19^ A transition state **TS2** (imaginary frequency 147.5 i cm^–1^) at 1.71 eV separates isomer **2a** by the other iso-isomer **2b** (Figure 4). The isomer **2b** can be considered a complex between CH_2_I^+^ and Cl with a distance of 3.28 Å between I and Cl atoms (Figure 3). Species **2b**, at 0.47 eV higher energy than isomer **2a**, can easily evolve into the final products Cl and CH_2_I^+^ with a threshold energy of only 0.11 eV. The picture obtained here for the Cl-loss channel is very similar to that found for the isomerization of geminal [ICH_2_I]^•+^ into iso-[CH_2_I–I]^•+^ radical ions and highlights the importance of halogen–halogen bond and iso-isomers, already studied both experimentally and theoretically in the case of neutral ICH_2_I and CICH_2_I species.^37,72,73^ The I-loss channel has been also theoretically investigated to compare it with the Cl-loss channel. In Figure 5 the optimized geometries of the species found on the [ClCH_2_I]^•+^ PES are shown, while the energy profile obtained for the isomerization mechanism of [ClCH_2_I]^•+^ into [CH_2_Cl−I]^•+^ is reported in Figure 6. In this case the direct C–I bond fission (on the left side of Figure 6) is the route energetically favored with respect to isomerization (right side of Figure 6) requiring an energy surplus of 0.76 eV to reach the **TS1b** (imaginary frequency 955.7 i cm^–1^ at 1.89 eV) and to produce the iso-isomer [CH_2_Cl–I]^•+^**3**. Isomer **3** is 1.36 eV above isomer **1**. Moreover, isomer **3** easily dissociates into CH_2_Cl^+^ + I through the transition state **TS2b** (imaginary frequency 378.6 i cm^–1^ at 1.37 eV) very close in energy to isomer **3** (Figure 6).

These findings clearly demonstrate that the isomerization of **1** to **3** is an unfavorable process with respect to the isomerization of **1** to **2a**. Therefore, likely only the iso-[CH_2_I–Cl]^•+^ radical cation is formed during the photofragmentation process. This is confirmed by the good agreement between A*E*_exp_(CH_2_Cl^+^) = 10.79 ± 0.01 eV and A*E*_th_(CH_2_Cl^+^) = 10.87 eV obtained by the direct C–I bond dissociation (Tables S1 and 2). The good agreement between these two values makes the isomerization process unlikely because in such a case the A*E*_th_ (CH_2_Cl^+^) would be significantly larger, i.e., 11.59 eV (Table S1). In any case, if the energy of the system should allow overcoming the barrier to **TS1b** to form isomer **3**, it rapidly fragments into CH_2_Cl^+^ and I trough **TS2b** due to the very low energy barrier (Figure 6). It is to be noted that the PIEC of the ion CH_2_Cl^+^ (*m*/*z* 49) in Figure 2 shows a change in the slope at about 11.2 eV. This is very close to the predicted ionization energy (11.237 eV) of the HOMO–2 orbital by the OVGF calculations. This orbital is an iodine lone pair type mixed with C–I bonding contributions and probably its removal opens a new channel for the CH_2_Cl^+^ formation.

In the case of I^+^ and ICl^+^ ions the A*E*_exp_ are 13.15 ± 0.19 and 15.01 ± 0.02 eV, respectively. These values are in quite disagreement with the adiabatic theoretical values of 12.83 eV (I^+^) and mostly of 14.01 eV (ICl^+^; Table 2) calculated in a barrierless fragmentation. The same procedure and level of *ab initio* calculations applied to [ICH_2_I]^•+^^20^ predicts appearance energies of I_2_^+^ and I^+^ in perfect agreement with the experiments (see Table S3 in the SI). These findings are quite intriguing and currently not completely clear. Further experiments and calculations, considering eventually spin–orbit effects, energy barriers, and excited states have to be undertaken to further investigate these observations.

## 5 Conclusions

In this work the dynamics of the Cl and I-loss channels from the geminal [ClCH_2_I]^•+^ radical cation has been explored. Four species were found on the potential energy surface of [ClCH_2_I]^•+^: **1**, **2a**, **2b**, and **3**. The most stable species is the geminal isomer [ClCH_2_I]^•+^**1** which can easily isomerize into the iso-isomer [CH_2_I–Cl]^•+^**2a**, at 1.15 eV higher energy and with the I–Cl halogen–halogen bond of 2.30 Å. This species can evolve into the higher energy isomer [CH_2_I···Cl]^•+^**2b**, a complex between CH_2_I^+^ and Cl with an I–Cl bond length of 3.28 Å. Isomer [CH_2_Cl–I]^•+^**3** has been also found on the PES, but this species seems to be kinetically unstable and quickly dissociates into CH_2_Cl^+^ + I. The measured appearance energies of the Cl- and I-loss channels, A*E*_exp_ (CH_2_I^+^) = 11.66 ± 0.03 eV and A*E*_exp_ (CH_2_Cl^+^) = 10.79 ± 0.01 eV, are in agreement with both present theoretical calculations (A*E*_th_ 11.46 and 10.87 eV, respectively) and previous experimental results (Table 2).^44^ These results validate our computational approach for these fragmentation channels. The fragmentation of [ClCH_2_I]^•+^ into I^+^ and ICl^+^ requires further work since the experimental data and theoretical prediction are not in agreement as well as the processes leading to CCl^+^ and CI^+^, whose A*E*_exp_ have been measured but not yet calculated. Further experiments and calculations, considering eventually spin–orbit effects, energy barriers and excited states, will be undertaken to further investigate these processes.

The observation that geminal halomethanes radical cation can isomerize into iso-dihalomethanes can be of interest because this process may be a driving force in the aerosol formation due to the possibility of these species to form clusters via halogen–halogen bond. This work predicts that the isomerization of the geminal [ClCH_2_I]^•+^ into iso-chloroiodomethane radical cation may occur. Now the process has to be proved experimentally.

## Supporting Information Available

Table S1 (electronic energies of the species investigated); Figure S1 (photoionization efficiency curves (PIECs) of the selected ions CCl ^+^ and CI^+^); Table S2 (vibrational frequencies of [ClCH_2_I]^•+^ cation); and Table S3 (A*E*_exp_ and A*E*_th_ of ions I_2_^+^, ICl^+^, and I^+^ from ClCH_2_I and ICH_2_I molecules) (PDF)
