# Supplementary material for: VUV Photofragmentation of Chloroiodomethane: The Iso-CH2I–Cl and Iso-CH2Cl–I Radical Cation Formation
Source: J Phys Chem A. 2020 Aug 12;124(37):7491–9. doi: 10.1021/acs.jpca.0c05754 (PMC8010789; doi:10.1021/acs.jpca.0c05754)
Supplement: Supplementary file 1 — jp0c05754_si_001.pdf [file jp0c05754_si_001.pdf]

Supporting Information

VUV Photofragmentation of Chloroiodomethane: The Iso-CH_2I-Cl and Iso-CH_2Cl-I Radical Cation Formation

Anna Rita Casavola,[1] *Antonella Cartoni,*[2,1]* *Mattea Carmen Castrovilli,*[1] *Stefano Borocci,*[3,4] *Paola Bolognesi,*[1] *Jacopo Chiarinelli*[1] *Daniele Catone,*[5] *and Lorenzo Avaldi*[1]

1) Institute of Structure of Matter-CNR (ISM-CNR), Area della Ricerca di Roma 1, 00015, Monterotondo, Italy

2) Department of Chemistry, Sapienza University of Rome, Rome, 00185, Italy

3) Department for Innovation in Biological, Agrofood and Forest Systems, University of Tuscia, Viterbo 01100, Italy

4) Institute for Biological Systems-CNR (ISB-CNR) Area della Ricerca di Roma 1, 00015 Monterotondo, Italy

5) Institute of Structure of Matter-CNR (ISM-CNR), Area della Ricerca di Tor Vergata, Rome, Italy

Corresponding Author: *to whom correspondence should be addressed:

antonella.cartoni@uniroma1.it

Tel. + 39 06 49913678

Table of Contents:

I. Electronic energies of the species investigated (Table S1)……………………………………S3

II. Photoionization efficiency curves (PIECs) of the selected ions CCl^+ and CI^+ (Figure S1)…...S4

III. Vibrational frequencies of $[ClCH_2I]^{\cdot+}$ cation (Table S2)………………………………………S5

IV. AE_{exp} and AE_{th} of ions I_2^+, ICl^+ and I^+ from $ClCH_2I$ and ICH_2I molecules (Table S3)……….S5

I. Electronic energies of the species investigated

Table S1. Electronic energy (E_e), zero point energy (ZPE), thermal correction (TC), electronic energy at 298 K ($E°_{298}$), enthalpy correction (EC) and enthalpy at 298 K ($H°_{298}$) in Hartree of all the stationary points investigated in the $ClCH_2I/ClCH_2I^{·+}$ system. In bold the species whose structures are shown in Figures 3 and 5 of the manuscript.

	E_e-CCSD(T,full)	ZPE (MP2)	E_0=E_e(full)+ZPE	TC (MP2)	$E°_{298}$	EC(MP2)	$H°_{298}$
[$ClCH_2I$] **N**	-793.8573754	0.028923	-793.8284524	0.003763	-793.8246894	0.004708	-793,8237444
[$ClCH_2I$]$^{·+}$ **(1)**	-793.5008914	0.028681	-793.4722104	0.003867	-793.4683434	0.004811	-793.4673994
**TS1**	-793.4364876	0.026205	-793.4102826	0.004155	-793.4061276	0.005099	-793.4051836
[CH_2I-Cl]$^{·+}$ **(2a)**	-793.4541996	0.024063	-793.4301366	0.004912	-793.4252246	0.005856	-793.4242806
**TS2**	-793.4342073	0.024849	-793.4093583	0.004424	-793.4049343	0.005368	-793.4039903
[CH_2I---Cl]$^{·+}$ **(2b)**	-793.4374961*	0.024643	-793. 4128531	0.00540	-793.4074531	0.006345	-793.4065081
[a]CH_2I^+	-333.7793546	0.024219	-333.7551356	0.002995	-333.7521406	0.003939	-333.7511966
Cl	-459.6535726		-459.6535726	0.001416	-459.6521566	0.002360	-459.6512126
**TS1b**	-793.427967	0.025330	-793.4026370	0.00387	-793.3987670	0.004815	-793.3978220
[CH_2Cl-I]$^{·+}$ **(3)**	-793.4475853	0.025350	-793.4222353	0.004661	-793.4175743	0.005605	-793.4166303
**TS2b**	-793.4485848	0.026766	-793.4218188	0.003707	-793.4181118	0.004651	-793.4171678
CH_2Cl^+	-498.5630017	0.025924	-498.5370777	0.002902	-498.5341757	0.003846	-498.5332317
[a]I	-294.8934684		-294.8934684	0.001416	-294.8920524	0.002360	-294.8911116
CH_2Cl	-498.870746	0.023457	-498.8472890	0.003379	-498.8439100	0.004323	-498.8429660
[b]I^+	-294.5115530		294.5115530	0.001416	-294.5101370	0.002360	-294.5091962
ICl^+	-754.2581919	0.001078	-754.2571139	0.002605	-754.2545089	0.003549	-754.2535649
[b]CH_2	-39.0749426	0.017745	-39.0571976	0.002854	-39.0543436	0.003798	-39.0534028

* with BSSE correction
[a] Reference 19 of the manuscript
[b] Reference 20 of the manuscript

II. Photoionization efficiency curves (PIECs) of the selected ions CCl^+, and CI^+

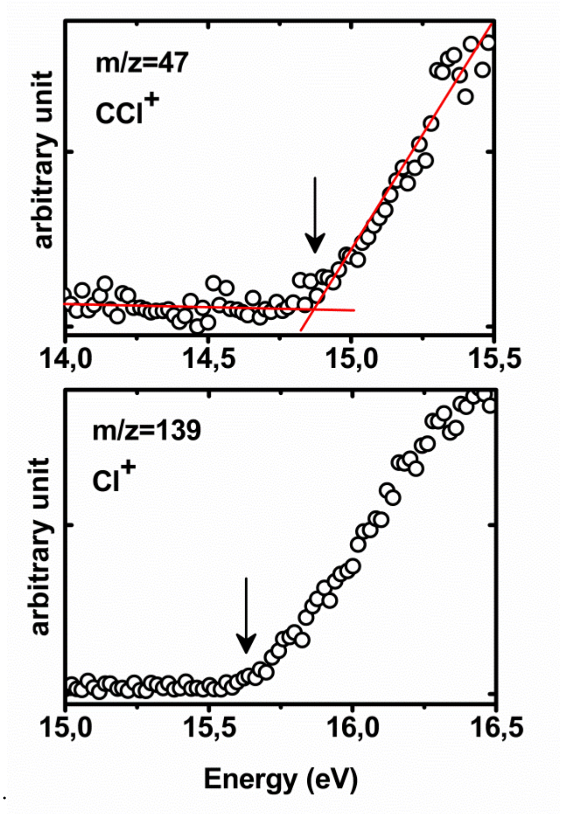

Figure S1. PIECs of the selected ions CCl^+ (m/z 47), and CI^+ (m/ 139). The fitted AE_{exp} values are indicated by black arrows and reported in Table 2 of the manuscript.

III. Vibrational frequencies of $[ClCH_2I]^{\cdot+}$ cation

Table S2. Vibrational frequencies (cm^{-1}) of $[ClCH_2I]^{\cdot+}$ cation in the ionic electronic ground state.

Mode character	This work MP2 (see theoretical methods)	DFT- B3LYP (without spin-orbit effect)[a]	DFT- B3LYP (with spin-orbit effect)[a]	MATI Experiments[b]
ν_1 C–H stretching (sym.)	3163	3126	3106	
ν_2 CH_2 scissoring	1468	1432	1420	1385
ν_3 CH_2 wagging	1230	1167	1175	1164
ν_4 C–Cl stretching	766	719	747	767
ν_5 C–I stretching	545	499	419	408
ν_6 Cl–C–I bending	147	160	112	114
ν_7 C–H stretching (asym.)	3278	3231	3206	
ν_8 CH_2 twisting	1116	1066	1089	1072
ν_9 CH_2 rocking	878	828	791	

[a] Reference 69 of the manuscript
[b] Reference 71 of the manuscript

IV. AE_{exp} and AE_{th} of ions I_2^+, ICl^+ and I^+ from $ClCH_2I$ and ICH_2I molecules

Table S3. AE_{exp} and adiabatic AE_{th} of ions I_2^+ , ICl^+ and I^+ from $ClCH_2I$ and ICH_2I molecules at 298 K.

	$ClCH_2I$		ICH_2I[a]	
**Ions (m/z)**	**AE_{exp} (eV)**	**AE_{th}(eV)**	**AE_{exp} (eV)**	**AE_{th}(eV)**
$I^+ + CH_2Cl/CH_2I$	13.15 ± 0.19	12.83	12.76± 0.03	12.76
$ClI^+/I_2^+ + CH_2$	15.01 ± 0.02	14.01	12.77 ± 0.02	12.71

[a] Reference 20 of the manuscript
